# Epithelioid Sarcoma of the Forearm Arising from Perineural Sheath of Median Nerve Mimicking Carpal Tunnel Syndrome

**DOI:** 10.1155/2009/595391

**Published:** 2009-04-14

**Authors:** Hiromasa Fujii, Kanya Honoki, Hiroshi Yajima, Akira Kido, Yasunori Kobata, Daisuke Kaji, Yoshinori Takakura

**Affiliations:** Department of Orthopedic Surgery, Nara Medical University, 840 Shijo-cho, Kashihara, Nara 634-8521, Japan

## Abstract

We report here a case of epithelioid sarcoma in the forearm of a 33-year-old male presenting with symptoms and signs of carpal tunnel syndrome originating from the direct involvement of the median nerve. Due to the slow growing of the tumor, the patient noticed the presence of tumor mass in his forearm after several months from the initial onset of the symptoms. Magnetic resonance imaging showed an 8 × 4 cm mass involving the median nerve in the middle part of the forearm, and histological analysis of the biopsy specimen revealed the diagnosis of epithelioid sarcoma. Radical surgical resection was performed in conjunction with adjuvant chemotherapy. The function of the flexors were restored by the multiple tendon transfers (EIP → FDS; ECRL → FDP; BrR → FPL; EDM → opponens) with superficial cutaneous branch of radial nerve transfer to the resected median nerve. The function of the affected hand showed excellent with the DASH disability/symptom score of 22.5, and both the grasp power and sensory of the median nerve area has recovered up to 50% of the normal side. The patient returned to his original vocation and alive with continuous disease free at 3.5-year follow-up since initial treatment.

## 1. Introduction

Epithelioid sarcoma (ES) is a rare distinctive soft tissue neoplasm with a predilection for the distal part of extremities, more often in upper than lower extremities, involving the subcutaneous tissue, tendon and fascia, and more often occurring in young adults and in the male than female [1–3]. It mostly presents as superficial nodules or ulcers; however deep-seated lesions can occur rarely. Although the clinical presentations are varying with a variety of physical-radiological features [4], it is extremely uncommon that ES involves the deep nerves [5, 6]. The initial treatment should be considered in a way of an aggressive wide resection with radical margins, sometimes in association with chemo- and radiotherapy [7]. It often recurs locally and also metastasizes usually in a few years time span preferably to the lung and sometimes to the lymph node. The reported long-term survival rates are around 50–60% [7].

We report a case of ES in the forearm presenting median nerve palsy as carpal tunnel syndrome clinically, and being treated by initial surgery in a form of radical resection of the bulk of tumor with finger flexors and median nerve as well. We describe the results of successful restoration of the finger flexors and median nerve function with multiple tendon transfers and nerve transfer from radial superficial cutaneous nerve branch after the radical resection of the tumor located in the forearm.

## 2. Case Report

A 33-year-old male presented with complaints of progressive sensory disturbance in median nerve area on his right hand since the Spring of 2005, and consulted a doctor in a district hospital then. Initial clinical assessment was the carpal tunnel syndrome, and he was conservatively treated with medication for a while with a wrist brace. However, median nerve palsy gradually progressed with weakening of *opponens pollicis* and *abductor pollicis brevis* muscles.

**Figure 1:**
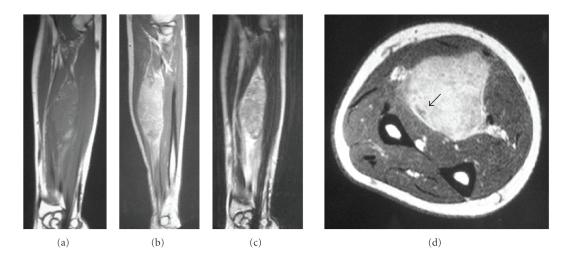
(a) Coronal T1-weighted, (b) T2-weighted, and (c) Gd enhanced magnetic resonance imaging shows a tumor measured approximately 8 × 4 × 3 cm. (d) Tumor encircles by the *flexor carpi radialis* (FCR), *flexor digitorum superficialis* (FDS), *flexor digitorum profundus* (FDP), and *flexor pollicis longus* (FPL), and pushes aside median nerve (arrow).

**Figure 2:**
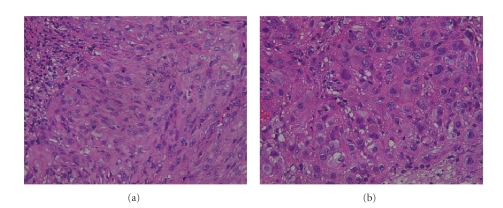
Histological appearance of open biopsy showing that tumor is consisted with malignant (a) spindle and (b) rounded cells with epithelioid features.

In October 2005, the patient himself eventually noticed the presence of tumor mass in the middle part of his forearm. Magnetic resonance imaging evaluation has been performed at that time, showing a tumor measured approximately 8 × 4 × 3 cm, encircled by the *flexor carpi radialis* (FCR), *flexor digitorum superficialis* (FDS), *flexor digitorum profundus* (FDP), and *flexor pollicis longus* (FPL) (see Figure 1). Open biopsy was subsequently performed, and histological appearance showed that tumor was consisted with malignant spindle cells with epithelioid features (see Figure 2), then he was referred to our institution afterward. The physical examination revealed an elastic hard tumor palpable in middle portion of his right forearm with remarkable Tinel's sign to the median nerve area. Positron emission topography showed very high uptake only at the middle part of right forearm, and no other abnormal uptake was detected. The computed tomography of the chest and laboratory data showed no other abnormalities. Immunohistochemical (IHC) analysis of the biopsy specimen was performed in our Pathology Department's laboratory, demonstrating the positive immunoreactivity to cytokeratin, CAM5.2, AE1/AE3, EMA, CD34, and smooth muscle action, in contrast negative immunoreactivity to desmin, S-100, Myc-2, MyoD, myogenin, myoglobin, and c-kit. These results of IHC with epithelial features from hematoxylin-eosin staining suggested the possible diagnosis of epithelioid sarcoma.

Chemotherapy consisted of two courses of ifosfamide (IFO; 12 g/m^2^/5 d) and adriamycin (ADM; 80 mg/m^2^/3 d) was administered; however tumor showed no regression of the size, therefore the surgery was performed in January 2006. Resection with curative wide surgical margin was achieved by en-block tumor mass resection in conjunction with surrounding tissues including median nerve and a part of ulnar artery, FDS, FCR, FDP, FPL, and palmaris longus (PL). After the resection, reconstruction of finger flexor was performed in a way of tendon transfers with *extensor indicus proprius* (EIP) to *flexor digitorum superficialis* (FDS); *extensor* carpi radialis longus (ECRL) to *flexor digitorum profundus* (FDP); brachioradialis (BrR) to *flexor pollicis longus* (FPL), and *extensor digiti minimi* (EDM) for *opponens* reconstruction. The superficial cutaneous branch of radial nerve was transferred to the distal end of the resected median nerve in order to recover the sensory deficit of median nerve under the sacrifice of sensory on dorsal side.

**Figure 3:**
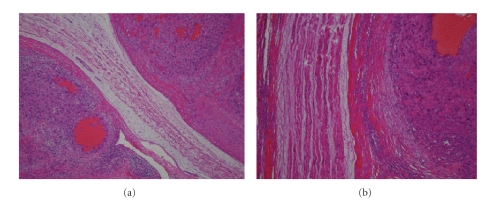
(a) Histological appearance of postoperative section showing that no tumor cell is infiltrated into the nerve, but (b) the pseudocapsule of the tumor is transitioning to the perineural sheath of the median nerve.

**Figure 4:**
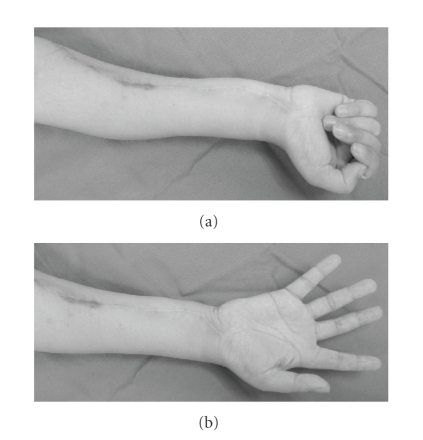
Photograph of right forearm on 2 years after operation. Grasp power of the affected hand has gained up to 50%, and the sensory of the median nerve area has also been recovered up to 50% of the unaffected side.

The macroscopic appearance of the resected tumor showed that the median nerve was directly involved in the tumor. Microscopically, no tumor cell was infiltrated into the nerve, but the pseudocapsule of the tumor was transitioning to the perineural sheath of the median nerve (see Figure 3), suggesting that epithelioid sarcoma could arise from nerve sheath as well as deep fascia or tendon sheath. The effect of preoperative chemotherapy showed Grade II of Huvos grading system [8]. Postoperative alternative chemotherapy consisted of a course of cisplatin (CDDP; 120 mg/m^2^/d) and adriamycin (ADM; 20 mg/m^2^/2 d) was administered, then rehabilitation has started. The fusion gene of SYT-SSX was not detected by reverse transcriptase-polymerase chain reaction in the specimen from definitive surgery. Thus, the final diagnosis was confirmed as epithelioid sarcoma. He has gained finger mobility and gotten back to his vocation within 3 months after the therapy. After 3 years of operation, the patient is alive with continuous disease free. The function of the affected hand showed DASH score of disability/symptom score of 22.5 and optional component score (sports/music or work optional module) of 50 (see Figure 4) [9]. Grasp power of the affected hand has gained up to 50%, and the sensory of the median nerve area has also been recovered up to 50% of the unaffected side.

## 3. Discussion

Epithelioid sarcoma (ES) is a rare malignant soft tissue neoplasm, usually slow and indolent growing, but very aggressive in occasion. Thus, the clinical behavior and outcome is unpredictable [10, 11]. In the series of Mayo clinic, 38% of the patients had at least one recurrence and 47% had metastatic lesions, then 27% of the patients died out of disease [11]. Although ES is one of the most common soft tissue sarcomas in the forearm, wrist and hand, soft tissue sarcomas themselves are very rare in these body parts [12]. In addition, ES is often difficult to diagnose because it may behave like a benign tumor both clinically and on imaging [4]. ES around the hand often mimics ganglion cyst [1] or other type of benign tumors like peripheral nerve sheath tumor, both possibly presenting the clinical symptoms compatible with carpal tunnel syndrome [6]. However, involvement and growth around a major peripheral nerve and presentation as a peripheral nerve sheath tumor are extremely rare [5]. In our case, due to the slow growing of the tumor, the patient noticed the mass presentation in his forearm after several months from the occurrence of symptoms mimicking with carpal tunnel syndrome, and MRI eventually revealed the tumor involvement of the median nerve.

Histologically, ES is often mistaken for benign entities as well as other type of malignancies including synovial sarcoma. The metaplastic conversion of mesenchymal cells to epithelia in a fashion that is not dissimilar to synovial sarcoma is a possible cause [1, 13]. The neoplastic cells generally coexpress keratin and vimentin, and often positive for CD34. In the series of Miettinen et al., the IHC profiles of 112 cases of ES including 88 typical and 24 variant (8 angiomatoid, 9 large cell/rhabdoid, and 7 fibroma-like) demonstrated that both typical and variant cases were found frequent expression of CD34, consistent expression of EMA and keratin [13]. Our case showed positive immunoreactivity to cytokeratin, CAM5.2, AE1/AE3, EMA, CD34. and smooth muscle actin, which are compatible to the diagnosis of ES.

Aggressive radical excision seems to be most effective in preventing local recurrence as well as the occurrence of distant metastasis. Halling et al. reported that no recurrence was observed in the patients treated with wide or radical resection with adjuvant therapy of either chemotherapy or radiotherapy [11]. However, it is very difficult to accomplish the radical or even wide resection with minimizing the functional loss for the lesion of forearm, wrist and hand. Thus, reconstruction of flexion function of the forearm remains a challenge to the reconstructive surgeon. With development of microsurgical techniques, the functional microsurgical muscle flap has been proved as a reliable technique in reconstructive surgery. Several reports depicted the usefulness of various muscle flaps for reconstruction of the upper extremity function [14–18]. However, the free-muscle flaps with microsurgical techniques are rather troublesome and may encounter the postsurgical complications including the vascular embolization. There have been several reports suggesting the usefulness of tendon transfers to reconstruct the flexion function after massive loss of function by tumor resection in the forearm [17, 18]. In general, reconstruction should be accomplished by the simplest means possibly; therefore tendon transfers should be considered as an option for reconstruction of hand function. In our case, the multiple tendon transfers were performed to restore the flexion function despite of sacrificing some extensor mechanisms, and resulted in good functional outcome with patient's satisfaction. An argue will arise on the transfer of the superficial cutaneous branch of the radial nerve, because the sensory of the dorsal side of the hand is sacrificed. Usually, the nerve graft from sural nerve for instance would be performed such a case. However, the recovery of the grafted nerve function will take a longer period for the massive defect. The advantage in our case was that it took only 6 months to recover the sensory of the hand which is feasible for the daily use.

The administration of chemotherapy and radiotherapy for epithelioid sarcomas is still controversial. We cannot determine the advantage of the chemotherapy consisted of IFO, ADM, and CDDP from our case, because of the inefficiency as a neoadjuvant and the very short followup duration. Several authors described that the use of chemotherapy and radiotherapy had no significant effect on the long-term survival [10, 11, 19]. However, further studies will be required to determine the benefit of chemotherapy as adjuvant, especially for a patient with advanced disease.
